# HA/CD44 Regulates the T Helper 1 Cells Differentiation by Activating Annexin A1/Akt/mTOR Signaling to Drive the Pathogenesis of EAP

**DOI:** 10.3389/fimmu.2022.875412

**Published:** 2022-05-26

**Authors:** Jing Chen, Jialin Meng, Xiaoling Li, Xiao Li, Yi Liu, Chen Jin, Li Zhang, Zongyao Hao, Xianguo Chen, Meng Zhang, Chaozhao Liang

**Affiliations:** ^1^Department of Urology, The First Affiliated Hospital of Anhui Medical University, Hefei, China; ^2^Institute of Urology, Anhui Medical University, Hefei, China; ^3^Anhui Province Key Laboratory of Genitourinary Diseases, Anhui Medical University, Hefei, China

**Keywords:** chronic prostatitis/chronic pelvic pain syndrome, CD44, hyaluronan, ANX A1, Th1 cells

## Abstract

CD44 partcipates in multiple inflammatory reactions. Here, we aimed to investigate the role of CD44 and the ligand, hyaluronan (HA), on chronic prostatitis/chronic pelvic pain syndrome (CP/CPPS) pathogenesis. We found that CD44 was universally expressed in CD4^+^ lymphocytes in the peripheral blood of CP/CPPS patients. After silencing CD44 expression or delivering 4-methylumbelliferone (4-MU), the pain severity and prostatic inflammation were significantly relieved. *In vitro* assay found that HA/CD44 was able to regulate T helper 1 (Th1) cells differentiation, the deficiency of which diminished experimental autoimmune prostatitis (EAP) susceptibility. Bioinformatic analysis suggested that after HA or 4-MU treatment, mTOR signaling was significantly altered, and these results were confirmed by subsequent Western blotting assay. Besides, mass spectrometry and co-immunoprecipitation assays found that CD44 was able to interact with Annexin A1 (ANX A1), and this kind of interaction stabilized ANX A1 protein and maintained the activation of Akt/mTOR pathway. Meanwhile, HA-treatment-enhanced prostatic inflammation, Th1 cell differentiation, and Akt/mTOR pathway activation were reversed after silencing the expression of ANX A1 using shANX A1-lentivirus. The present study systematically investigates the functional role of HA/CD44 in CP/CPPS and identifies novel mechanisms for HA/CD44 promoting Th1 cell differentiation. Targeting the HA/CD44/ANX A1/Akt/mTOR signaling represents novel potential therapeutic strategies for patients with CP/CPPS.

## 1 Introduction

As an epidemic genitourinary disease in young and middle-aged men, the incidence of chronic prostatitis/chronic pelvic pain syndrome (CP/CPPS) ranges from 8.4% to 14% (1–3). The clinical symptoms of CP/CPPS patients mainly include a sequence of syndromes (4), which significantly affect the life quality of CP/CPPS patients (5, 6). Till now, the pathogenesis of CP/CPPS is unclear; increasing evidence indicates that the abnormalities of immune systems might play a considerable role (7). The revelation of the potential mechanism of CP/CPPS by in-depth study is urgently important for promoting the clinical treatment of CP/CPPS patients. Past studies had identified the infiltration of a series of immune-related cells, such as neutrophils and T lymphocytes, in the local prostate region of CP/CPPS patients (8, 9). As indicated in previous studies, the experimental autoimmune prostatitis (EAP) model shares similar features with human CP/CPPS patients in pelvic pain, inflammation, inflammatory infiltration, and elevated pro-inflammatory cytokines (10, 11). The major type of lymphocytes infiltrating around prostate is T helper 1 (Th1) cells in EAP mouse model (10, 11). Notably, previous study proved that NOD/LtJ non-obese diabetic (NOD) mice with interferon-gamma (IFN-γ)-deficiency (unable to mount normal Th1 responses) were unable to form prostatic inflammation (11). These discoveries confirmed the differentiation proportion of Th1 cells partly determining the initiation and progression of chronic prostatitis. However, the potential mechanisms of how Th1 cells proportion increased during the pathogenesis of CP/CPPS are still uncertain, and more in-depth studies are necessary.

CD44, expressed on the membrane of lymphoid and non-lymphoid cells, takes part in multiple physiological reactions, including homing, activation, and proliferation of lymphocyte cells (12). Many studies suggested that CD44 deficiency could diminish the severity of immune-mediated diseases, such as arthritis (13) and atherosclerosis (14). Studies also revealed that Th1 and T helper 17 (Th17) cells differentiation was diminished on CD44-deficient mice (15, 16). The major ligands of CD44 are hyaluronan (HA) and osteopontin. Inhibiting the HA synthesis reduces T-cells migration and modifies the polarization of T cells; in detail, the polarization of T cells is biased toward T helper 2 (Th2) cells and away from Th1/Th17 cells (17, 18). Although CD44 and its ligand, HA, play a vital role in inflammatory diseases, no relevant studies were conducted for the functional role of the HA/CD44 axis in Th1 cells differentiation regulation, even for the implication of CP/CPPS.

In this research, we found that CD44 expression in EAP mice’s prostate tissues was significantly increased compared with that of the control mice. We reflected the pivotal role of the HA/CD44 axis in Th1 cells differentiation and prostatic inflammation genesis. In-depth mechanism studies suggested that CD44 promotes Th1 cells differentiation partly through the HA-promoted CD44 and Annexin A1 (ANX A1) interaction and then further activation of downstream Akt/mTOR pathway. Therefore, targeting HA/CD44-ANX A1-Akt/mTOR cascade pathway could result in the decrease in Th1 cells differentiation and the relieving of severe inflammation and pain among CP/CPPS patients.

## 2 Materials and Methods

### 2.1 Reagents and Antibodies

The following manufacturer’s information of reagents and antibodies was included: complete Freund’s adjuvant (CFA; Sigma-Aldrich, St Louis, MO, USA), the commercial enzyme-linked immunosorbent assay (ELISA) kit for interleukin-17 (IL-17, cat#E-EL-M0047c; Elabscience Biotechnology, TX, USA), IFN-γ (cat#E-EL-M0048c; Elabscience Biotechnology, TX, USA), and interleukin-1β (IL-1β, cat#E-EL-M0037c; Elabscience Biotechnology, TX, USA), anti-mouse CD4-FITC (cat#553047; BD Biosciences, NJ, USA), CXCR3-PE (cat#562152; BD Biosciences, NJ, USA), IFN-γ-PE (cat#562020; BD Biosciences, NJ, USA), anti-CD3e (10 μg/ml, cat#BE0001-1; Bio X Cell, NJ, USA), anti-CD28 (10 μg/ml, cat#BE0015-1; Bio X Cell, NJ, USA), interleukin-12 (IL-12, 10 μg/ml, cat#CM39; Novoprotein, Shanghai, China), interleukin-2 (IL-2, 10 μg/ml, cat#CK24; Novoprotein, Shanghai, China), anti-recombinant hyaluronan binding protein 2 (anti-HABP2, 1:200, cat#AF9083; Affinity Biosciences, OH, USA), anti-osteopontin (1:200, cat#AF0227; Affinity Biosciences, OH, USA), anti-mTOR (1:1,000, cat#2983; Cell Signaling Technology, MA, USA), anti-phospho-mTOR (1:1,000, cat#5536; Cell Signaling Technology, MA, USA), anti-Akt (1:1,000, cat#4691; Cell Signaling Technology, MA, USA), anti-phospho-Akt (1:1,000, cat#13038; Cell Signaling Technology, MA, USA), anti-CD44 (1:1,000, cat#GTX628472; GeneTex, CA, USA), anti-ANX A1 (1:1,000, cat#GTX101070; GeneTex, CA, USA), and β-actin (cat#AF0501; Elabscience Biotechnology, TX, USA).

### 2.2 Mice Preparation

The EAP mice models were established with NOD male mice that were purchased from Nanjing Institute of Biomedical, Nanjing University, China. All experiments were conducted under the Guidelines for the Ethical Conduct in the Care and Use of Animals from the Animal Center of Anhui Medical University (approval no. LLSC201800488).

### 2.3 The EAP Mice Models Establishment and Grouping and Pelvic Pain Symptoms Evaluation

We prepared the immunoreactive reagent by fully emulsifying the prostate antigen, which was obtained from grinding prostate tissues of Sprague–Dawley (SD) rats, and the equal volume of CFA [19]; 0.15 ml (300 μg/mouse) of the immunoreactive reagent or saline solution was used to subcutaneously immunize at different parts, such as the base of the tail and hind footpad, of male NOD mice on days 0 and 28 [19]. The mice model experiments were mainly divided into two steps. First, the EAP mice models were established to perform RNA-seq analysis and verify the main ligands of CD44, for instance, HA and osteopontin. Second, based on the EAP mice models, we verified the role of CD44 and its ligands in EAP. To confirm the function of CD44, we constructed the mice model groups with and without CD44 knockout; four groups were divided by randomly assigning those male NOD mice (n = 7 in each group) (1): the Ctrl + NC group, NC-adeno-associated virus (AAV) was injected into mice *via* the tail vein 1 week before the two subcutaneous immunization of saline solution (2); the Ctrl + sh*Cd44* group, mice were injected with sh*Cd44*-AAV *via* the tail vein 1 week before the two subcutaneous immunization of saline solution (3); the EAP + NC group, mice were injected with NC-AAV *via* the tail vein 1 week before the two subcutaneous immunization of immunoreactive reagent; and (4) the EAP + sh*Cd44* group, mice were injected with sh*Cd44*-AAV *via* the tail vein 1 week before the two subcutaneous immunizations of immunoreactive reagent. Then, the function of HA was also evaluated by the mice model, and the grouping and experiment implementation were carried out in accordance with our previous study [19]. On the 14th day after the second immunization, we tested the response frequency of mice to pelvic stimuli for all filaments before sacrificing them. The positive reaction to pain was considered when the following three reactions occurred: (a) sharp abdomen contraction, (b) immediate licking or scratching of the area stimulated by filaments, or (c) jumping.

### 2.4 Hematoxylin–Eosin Staining, Immunohistochemistry Assay, and ELISA Analysis

The prostate tissues isolated from the mice were fixed with 4% formaldehyde solution and then embedded in the paraffin. Five-micrometer-thick sections of the prostate tissue were used for HE and immunohistochemistry (IHC) analyses. The detailed experimental procedures for HE and IHC assay have been described in our previous literature [19]. The inflammation grade was divided into four grades, which were from 0 to 3: 0, no inflammation; 1, mild but definite perivascular cuffing with mononuclear cells; 2, moderate perivascular cuffing with mononuclear cells; and 3, marked perivascular cuffing, hemorrhage, and numerous mononuclear cells in the parenchyma. The concentrations of IFN-γ, IL-1β, and IL-17 in mice sera were detected by ELISA assay following the manufacturer’s instructions.

### 2.5 Isolation of Th1 Cells, Flow Cytometry Analysis, and RNA Sequencing

#### 2.5.1 Isolation of Th1 Cells and Flow Cytometry Analysis

The single-cell suspension was prepared according to our previous studies [20]. Th1 cells were sorted out by the FACSCalibur flow cytometer (BD Biosciences, Franklin Lakes, USA) after the cells of mice spleens were incubated with anti-mouse CD4-fluorescein isothiocyanate (FITC) and CXCR3-phycoerythrin (PE) antibodies at room temperature for 1 h, and the purity of the sorted cells was over 95%. To analyze Th1 cells proportion, the cells were stained with anti-mouse CD3-allophycocyanin (APC), CD4-FITC, and IFN-γ-PE antibodies.

#### 2.5.2 RNA Sequencing and Bioinformatic Analyses

RNA was extracted from Th1 cells and prostate tissues of mice by standard extraction methods. Then, the quality control on the RNA samples was conducted from three aspects. dNTP was used as the raw source for the second cDNA strand synthesis in the DNA polymerase I system. The libraries received amplification and purification of the PCR products with AMPure XP beads. The NEBNext^®^ Ultra™ RNA Library Prep Kit for Illumina^®^ was employed to construct the libraries. RNA sequencing of the qualified libraries was conducted by the Illumina platform.

### 2.6 Isolation and *In Vitro* Differentiation of Naive CD4^+^ T Cells

The single-cell suspension was co-cultured with Biotin-Antibodies Cocktail, Anti-Biotin MicroBeads, and CD44 MicroBeads, and unlabeled cells were collected through LS Column according to protocols of Naive CD4^+^ T Cell Isolation Kit (cat#130-104-453; Miltenyi Biotec, CA, USA). To detect whether HA affects Th1 cells differentiation, 5 μg/ml of HA was added in the 1640 medium. According to prior studies, the intervention groups were treated with 10 μM MK-2206 (the inhibition of Akt), 20 μg/ml everolimus (the inhibition of mTOR), or same volume of phosphate buffered saline (PBS) [20, 21]. Following the protocols of the manufacturer, naive CD4^+^ T cells were also transfected with sh*Anxa1*-lentivirus or NC-lentivirus (Hanbio Biotechnology, Shanghai, China). The subsequent experiments were carried out in a culture period of 4 days.

### 2.7 Immunoprecipitation, Silver Staining, and Mass Spectrometry

One milliliter of cell immunoprecipitation lysis buffer was used to lysis the cells followed by centrifuging to collect the supernatant. Twenty microliters of A/G Agarose sugar beads washed twice by PBS was added to the tube containing the protein solution for incubating at 4°C for 2 h, following by transferring an equal volume of the supernatant to two new 1.5-ml EP tubes, which were numbered as A and B, respectively. Tubes A and B were added with IgG (1 μg), CD44 (1 μg), or ANX A1 (1 μg), respectively, and incubated overnight at 4°C on a vertical rotator. After washing twice with lysis buffer, 50 μl of 2× sodium dodecyl sulfate (SDS) loading was added to each EP tube and boiled in boiling water for 15 min for subsequent experiments. Ten microliters of the protein samples collected from the IP experiment was added to 10% SDS-polyacrylamide gels at a low voltage of 60 V. A series of steps for the gels, such as fixation, rinsing, immobilization sensitization, silver staining, and color rendering, were conducted according to the protocols of PierceTM Silver Stain for Mass Spectrometry (cat#24600; Thermo Fisher Scientific, MA, USA). The different protein bands were cut for subsequent mass spectrometry.

Before mass spectrometry, silver-stained samples were enzymatically hydrolyzed and desalted according to the previous study [24]. After the peptide samples were dissolved in nano-high-performance liquid chromatography (nano-HPLC) buffer, the samples were separated by EASY-nLC1200 (Thermo Fisher Scientific, MA, USA) and then analyzed by a Q-Exactive mass spectrometer (Thermo Fisher Scientific, MA, USA).

### 2.8 Western Blotting

The expression levels of CD44, ANX A1, p-Akt, Akt, p-mTOR, and mTOR were detected by Western blotting. SDS-polyacrylamide gels (8%) were used to separate p-Akt, Akt, p-mTOR, and mTOR proteins, and 10% SDS-polyacrylamide gels were used to separate CD44 and ANX A1 protein, followed by transferring of the proteins to NC membranes (Bio-Rad, Hercules, CA, USA).

### 2.9 Statistical Analysis

Mean value ± standard deviation (mean ± SD) was used to describe the data. Student’s t-test and one-way ANOVA test were used for statistical analysis. The p-value <0.05 was considered as statistically significant. All these analyses were performed by R-4.0.2 software and GraphPad Prism 6.0 (La Jolla, CA, USA).

## 3 Results

### 3.1 Identification and Validation of the Key Molecule-CD44 in CP/CPPS

**Figure 1:**
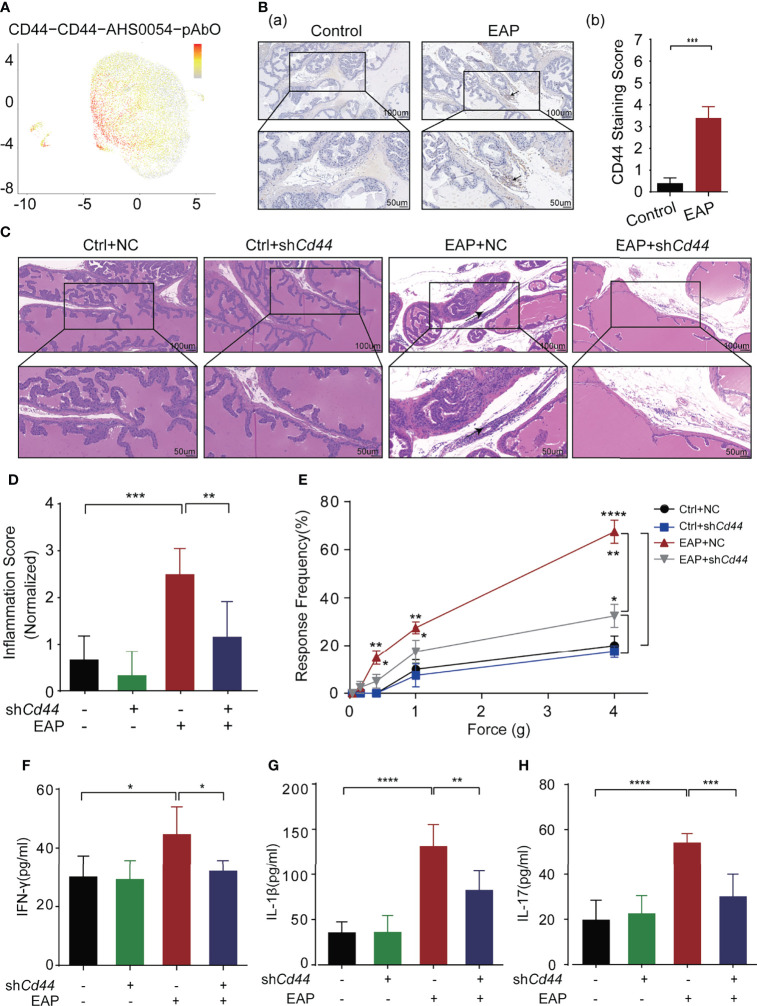
Identification and validation of key molecule-CD44 for CP/CPPS. **(A)** Single-cell gene sequencing showed that CD44^+^T cells accounted for more than 93% of the total T lymphocytes. **(B)** The expression of CD44 in prostate tissues of mice between control and EAP groups. **(C)** Analysis of histological difference by HE staining. **(D)** Corresponding histopathological score and data analysis according to data in Panel **(C)**. **(E)** Tactile allodynia development in NOD mice from those four groups. **(F)** The secretion levels of IFN-γ in serum of NOD mice from those four groups. **(G)** The secretion levels of IL-1β in serum of NOD mice from those four groups. **(H)** The secretion levels of IL-17 in serum of NOD mice from those four groups. ****p < 0.0001, ***p < 0.001, **p < 0.01, *p < 0.05.

In our recent studies, we discovered several interesting biomarkers that were able to reflect the inflammatory status of mice prostate (unpublished). Of these markers, we focused on CD44, which was expressed on 93% of CD4^+^ T lymphocytes, identified by single-cell multi-omics sequencing analysis (**Figure 1A**) (8) and mainly expressed on the infiltrated lymphocytes instead of the local prostate’ epithelial cells (**Figure 1B**; **Supplementary Figure 1**).

To validate the biological role of CD44 in CP/CPPS, we injected sh*Cd44*-AAV into the EAP mouse through the tail vein. The expression levels of CD44 in CD4^+^T cells from mice were detected by Western blotting (WB). The results of WB showed that sh*Cd44* could significantly decreased the expression of CD44 (**Supplementary Figure 2C**). Compared with mice in the EAP + NC group, the immunocytes infiltration to the prostatic interstitium was diminished in the EAP + sh*Cd44* group (*p* < 0.01, **Figures 1C** **, D**). Consistently, the tactile allodynia frequency of mice in the EAP + sh*Cd44* group was significantly reduced compared with that of the EAP + NC group at the force of 0.4, 1.0, and 4.0 g (*p* < 0.05, **Figure 1E**). Besides, the levels of IFN-γ (*p* < 0.05, **Figure 1F**), IL-1β (*p* < 0.01, **Figure 1G**), and IL-17 (*p* < 0.001, **Figure 1H**) in mice of the EAP + sh*Cd44* group were significantly reduced compared with mice in EAP + NC group. Taken together, the results from **Figure 1** and **Supplementary Figure S1** suggest that CD44 involves in the pathogenesis of CP/CPPS.

### 3.2 Identification and Validation of Key Ligand of CD44, HA

**Figure 2:**
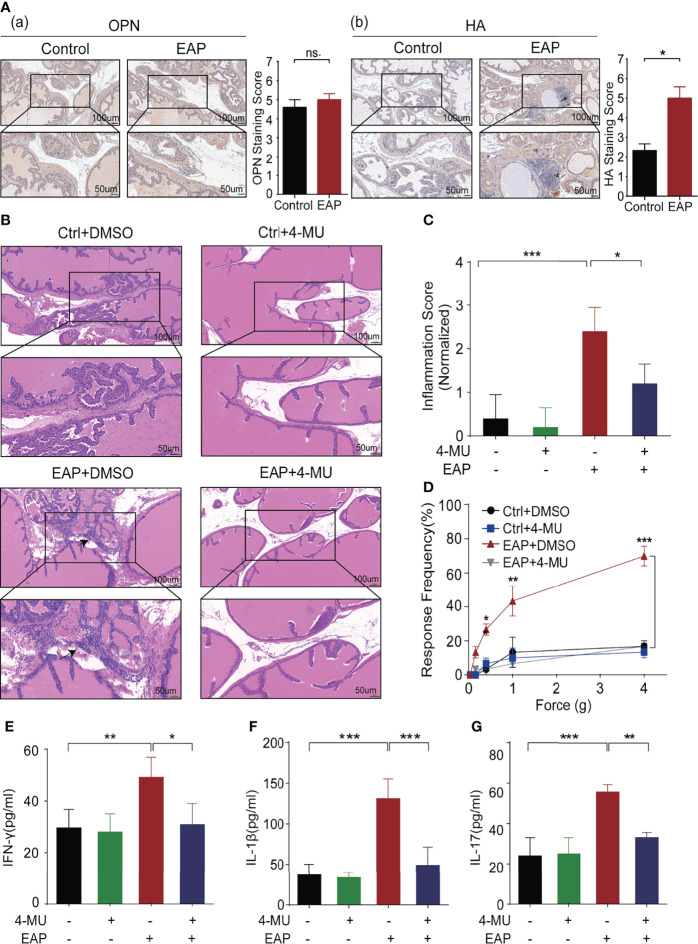
Identification and validation of key ligand of CD44, HA, for CP/CPPS. **(A)** The expression of important ligands of CD44, OPN, and HA, in prostate tissues of mice between the control and EAP groups. **(B)** Analysis of histological difference by HE staining. **(C)** Corresponding histopathological score and data analysis according to data in Panel **(B)**. **(D)** Tactile allodynia development in NOD mice from those four groups. **(E)** The secretion levels of IFN-γ in serum of NOD mice from those four groups. **(F)** The secretion levels of IL-1β in serum of NOD mice from those four groups. **(G)** The secretion levels of IL-17 in serum of NOD mice from those four groups. ***p < 0.001, **p < 0.01, *p < 0.05.

The main ligands of CD44 are HA and osteopontin (OPN). We previously proved that HA is related to the severity of symptoms in CP/CPPS patients, and targeting HA synthesis by 4-MU significantly relieved the prostatic inflammation (19). IHC reflected that HA was higher expressed in prostate tissues derived from EAP mice as compared with the control, while we failed to identify obvious differences for OPN between these two groups (**Figure 2A**; **Supplementary Figure 2A**).

To further validate the biological function of HA in CP/CPPS, we intraperitoneally injected 4-MU (200 mg/kg/day), an inhibitor that could block the synthase of HA, to the EAP mice daily from day 0. Comparing with the mice in the EAP + dimethyl sulfoxide (DMSO) group, we found that the immunocytes infiltration to the prostate was significantly decreased as compared with the mice in the EAP + 4-MU group (*p* < 0.05, **Figures 2B** **, C**). Consistently, the response frequency of tactile allodynia in the EAP + 4-MU group was significantly lower at the force of 0.4, 1.0, and 4.0 g, than that of the EAP + DMSO group (all *p* < 0.05, **Figure 2D**). Furthermore, we also found the declined expression of IFN-γ (*p* < 0.05, **Figure 2E**), IL-1β (*p* < 0.001, **Figure 2F**), and IL-17 (*p* < 0.01, **Figure 2G**) in mice of the EAP + 4-MU group than the EAP + DMSO group. Taken together, the results from **Figures 1**, **2** suggest that HA/CD44 axis plays a critical role in the pathogenesis of chronic prostatitis.

### 3.3 HA/CD44 Axis Drives EAP Initiation by Enhancing the Th1 Cell Differentiation

**Figure 3:**
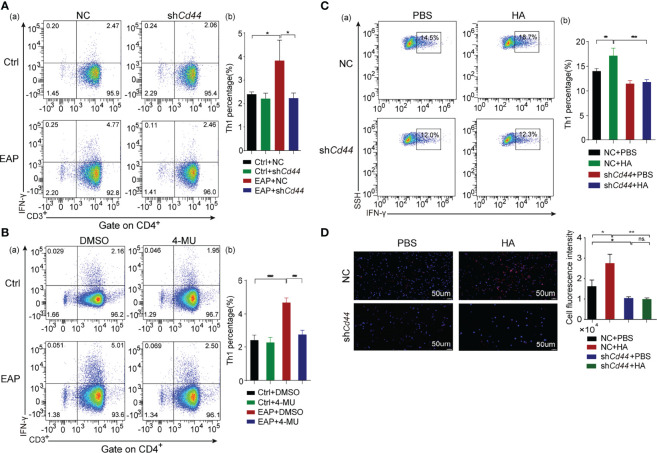
HA/CD44 axis plays an important effect on the occurrence of EAP by promoting Th1 cells differentiation. **(A)** The percentage of Th1 cells in splenic lymphocytes of immunized mice from the Ctrl + NC, the Ctrl + sh*Cd44*, the EAP + NC, and the EAP + sh*Cd44* groups by flow cytometry (a, cell flow cytometry diagram and b, corresponding data analysis according to data in a). **(B)** The percentage of Th1 cells in splenic lymphocytes of immunized mice from the Ctrl + DMSO, the Ctrl + 4-MU, the EAP + DMSO, and the EAP + 4-MU groups by flow cytometry (a, cell flow cytometry diagram and b, corresponding data analysis according to data in a). **(C)** The differentiation ratio of Th1 cells in the *in vitro* differentiation experiment by flow cytometry (a, cell flow cytometry diagram and b, corresponding data analysis according to data in a). **(D)** The differentiation ratio of Th1 cells (IFN-γ) in the *in vitro* differentiation experiment by immunofluorescence. ns, Not Significant. ***p < 0.001, **p < 0.01, *p < 0.05.

Since Th1 cells have been proven to be indispensable during the pathogenesis of CP/CPPS (20), we investigated the regulatory effect of HA/CD44 on Th1 cell differentiation. We found that the proportion of Th1 cells was lower in the sh*Cd44* or 4-MU treatment subgroup, compared with the vector or DMSO treatment set, respectively (**Figures 3A** **, B**). In *in vitro* assay, compared with PBS treatment, HA significantly promoted the naive CD4^+^T cells derived from mice in the NC-AAV group differentiated into Th1 cells, while the differentiation was arrested after knocking down CD44 expression (**Figures 3C** **, D**). Taken together, the results from **Figure 3** suggest that HA/CD44 axis takes part in the pathogenesis of CP/CPPS through regulating Th1 cell differentiation.

### 3.4 HA/CD44 Axis Activated the Akt/mTOR Pathway to Promote Th1 Cells Differentiation

**Figure 4:**
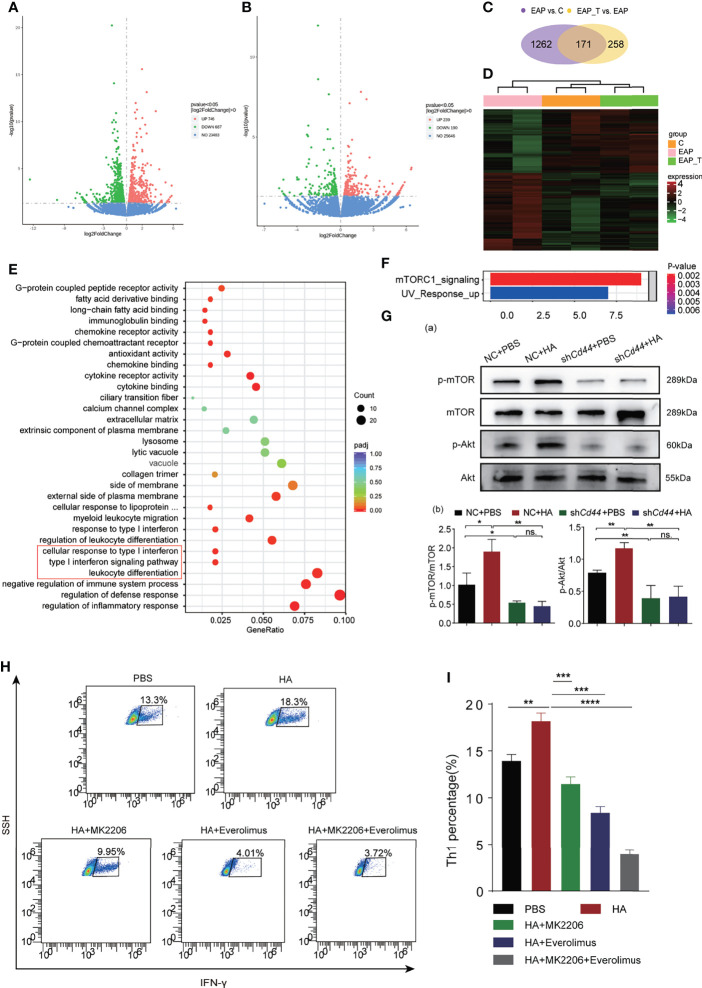
HA/CD44 axis activated the Akt/mTOR pathway to promote Th1 cells differentiation. **(A)** Differentially expressed genes of Th1 cells from NOD mice among the Ctrl + DMSO and EAP + DMSO groups. **(B)** Differentially expressed genes of Th1 cells from NOD mice among the EAP + DMSO and EAP + 4-MU groups. **(C)** Mutual differentially expressed genes among EAP + DMSO vs. Ctrl + DMSO and EAP + 4-MU vs. EAP + DMSO. **(D)** Heatmap of Th1 cells from NOD mice among the Ctrl + DMSO, EAP + DMSO, and EAP + 4-MU groups. **(E)** The differential genes enriched pathways by the analysis of GO. **(F)** The differential genes enriched pathways by the analysis of KEGG. **(G)** The expression levels of p-mTOR, mTOR, p-Akt, and Akt of Th1 cells in the *in vitro* differentiation experiment among NC + PBS, NC + HA, sh*Cd44* + PBS, and sh*Cd44* + HA groups. **(H)** The differentiation ratio of Th1 cells in the *in vitro* differentiation experiment among PBS, HA, HA + MK2206, HA + Everolimus, and HA + MK2206 + Everolimus groups by flow cytometry. **(I)** Corresponding data analysis according to data in **(H)**. ns, Not Significant. ****p < 0.0001, ***p < 0.001, **p < 0.01, *p < 0.05.

To study the mechanism of how the HA/CD44 axis regulates Th1 cell differentiation, we sorted Th1 cells from mice in the Ctrl + DSMO, EAP + DMSO, and EAP + 4-MU groups, respectively, and performed RNA-sequencing analysis. Differentially expressed genes (DEGs) were identified with a threshold *p*-value <0.05 (**Figures 4A** **, B**). A total of 171 overlapped DEGs were collected after intersecting DEGs from the EAP vs. control group and EAP + 4-MU vs. EAP group (**Figures 4C** **, D**). These DEGs were mainly enriched in immune-related pathways, such as regulation of inflammatory response, regulation of defense response, negative regulation of immune system process, and leukocyte differentiation (**Figure 4E**). Notably, Hallmark pathway analysis found that after HA stimulation, mTOR signaling was activated (**Figure 4F**), which has been validated in Th1 cells from mice of the control and EAP groups by WB (**Supplementary Figure 2B**). Subsequent Western blotting assay found that the p-mTOR and p-Akt expression in naive CD4^+^T cells was increased after HA stimulation, while these effects were reversed after knocking down CD44 expression (**Figure 4G**). Akt inhibitor (MK-2206) or mTOR inhibitor (everolimus) could reverse the promotion of Th1 differentiation of HA/CD44 axis in the *in vitro* Th1 differentiation experiment (**Figures 4H** **, I**). Taken together, the results from **Figure 4** suggest that HA/CD44 axis is partly through activating the AKT/mTOR signaling to increase Th1 cell differentiation and thus drives the pathogenesis of CP/CPPS.

### 3.5 HA Promotes the Interaction Between CD44 and ANX A1 to Activate Akt/mTOR Signaling

**Figure 5:**
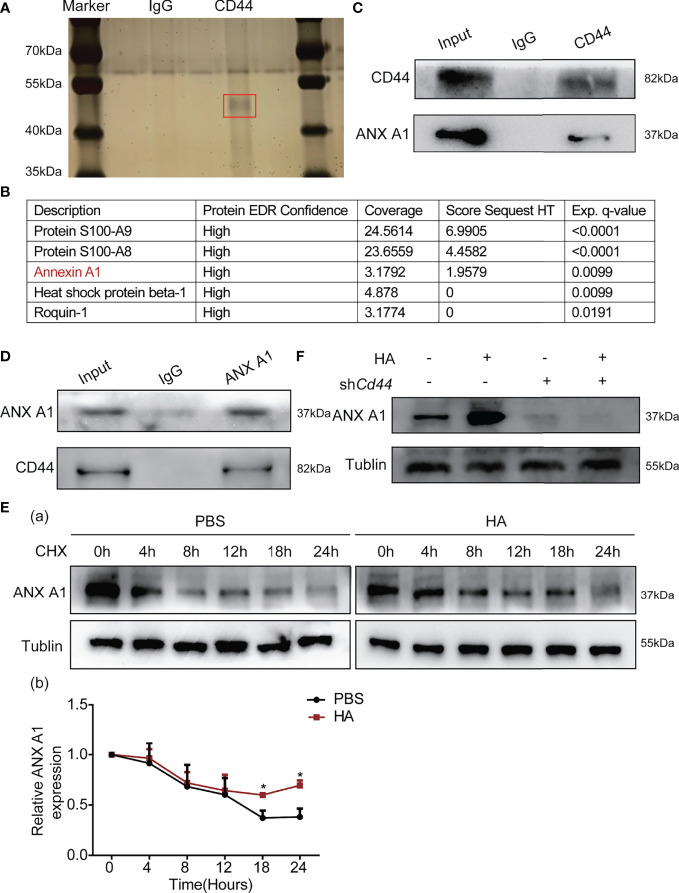
HA/CD44 axis promoted the expression and the stability of ANX A1 at the protein level. **(A)** Silver staining detected related proteins compared with red marker showing the differential band. **(B)** The results of mass spectrometry. **(C)** WB assay verified that HA/CD44 directly interacted with ANX A1. **(D)** WB assay verified that the interaction between ANX A1 and CD44 at the protein level through pulling-down ANX A1. **(E)** The stability of ANX A1 with or without CHX in the *in vitro* Th1 cells differentiation experiment with or without the existence of HA was detected by WB assay (a, the protein bands and b, corresponding data analysis according to data in a). **(F)** The expression of ANX A1 in the *in vitro* Th1 cells differentiation experiment with or without the existence of HA was detected by WB assay. *p < 0.05.

To understand how HA/CD44 activates the Akt/mTOR signaling, we employed mass spectrometry by pull-down CD44 after naive CD4^+^T cells stimulated by HA (**Figure 5A**). Among these potential candidates, we focused on ANX A1, which is expressed on various immunocytes, as one of the new candidates that could potentially interact with CD44 (**Figure 5B**). Subsequently, IP assay by pull-down CD44 or ANX A1 protein proved that CD44 was able to interact with ANX A1 (**Figures 5C** **, D**). Then, the CHX experiments confirmed that the ANX A1 protein stability was enhanced after the integration of HA and CD44 (**Figure 5E**). WB assay indicated that ANX A1 expression for the naive CD4^+^T cells transfected with NC-AAV was significantly increased after the stimulation of HA as compared with the naive CD4^+^T cells transfected with sh*Cd44*-AAV (**Figure 5F**).

**Figure 6:**
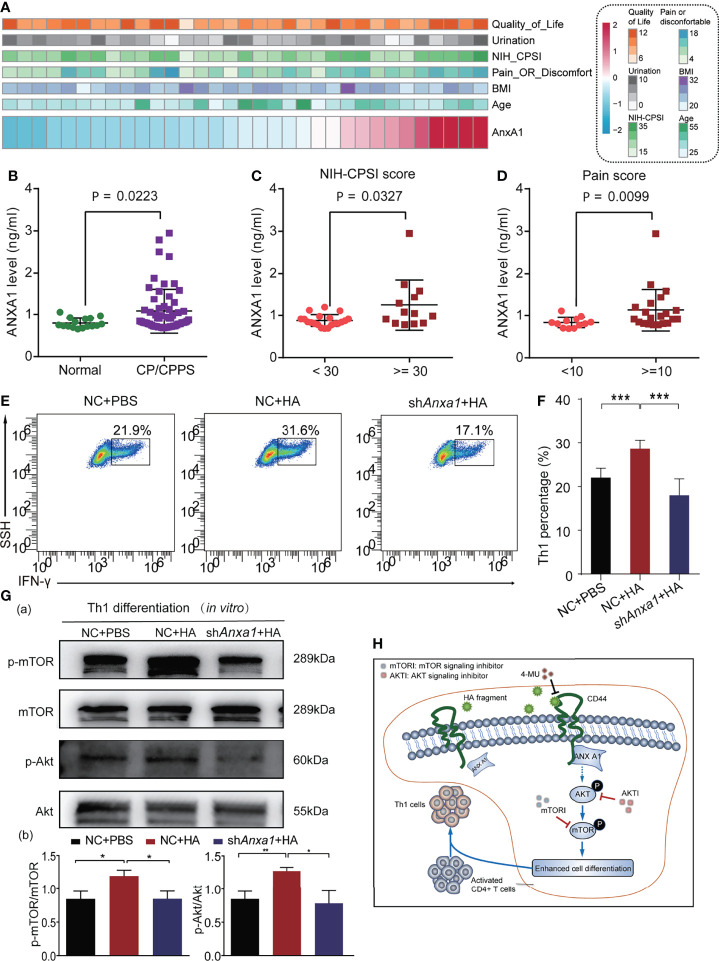
HA/CD44 axis activated the Akt/mTOR pathway by promoting the expression of ANX A1, which was increased in CP/CPPS patients to promote Th1 cells differentiation. **(A)** Heatmap revealing the correlation between ANX A1 and several clinical features. **(B)** ANX A1 increased in CP/CPPS patients. **(C)** ANX A1 increased in severe NIH-CPSI scores. **(D)** ANX A1 increased in severe pain symptoms. **(E)** The differentiation percentage of Th1 cells in the *in vitro* differentiation experiment among NC + PBS, NC + HA, and sh*Anxa1* + HA groups. **(F)** Corresponding data analysis according to data in **(E, G)** The expression levels of p-mTOR, mTOR, p-Akt, and Akt of Th1 cells in the *in vitro* differentiation experiment among NC + PBS, NC + HA, and sh*Anxa1* + HA groups. **(H)** The possible mechanism of HA/CD44 promoting Th1 cells differentiation. ***p < 0.001, **p < 0.01, *p < 0.05.

We collected serum samples from 64 CP/CPPS-like patients and 16 healthy controls to analyze the clinical significance of ANX A1. The demographic parameters of the CP/CPPS patients are recorded in **Supplementary Table 1**. The distribution of ANX A1 expression and clinical parameters of 64 CP/CPPS patients is displayed in **Figure 6A**. As compared with healthy controls, CP/CPPS-like patients contained a higher level of ANX A1 (*p* = 0.0223, **Figure 6B**). Besides, CP/CPPS patients with severe clinical symptoms expressed a higher level of ANX A1 (NIH-CPSI score: <30 vs. ≥30, *p* = 0.0327, **Figure 6C**; pain score: <10 vs. ≥10, *p* = 0.0099, **Figure 6D**).

We further explored the biological role of ANX A1 on Th1 differentiation cells in *in vitro* study. Naive CD4^+^ T cells sorted from mice were transfected with shANX A1-lentivirus or NC-lentivirus to knock down the ANX A1 expression. After naive CD4^+^ T cells were transferred with sh*Anxa1*, the expression levels of ANX A1 in naive CD4^+^ T cells were detected by WB. The results of WB showed that sh*Anxa1* could significantly decrease the expression of ANX A1 in naive CD4^+^ T cells (**Supplementary Figure 2D**).The stimulation effects of HA on Th1 cells differentiation were reversed after transfecting with sh*Anxa1*-lentivirus (**Figures 6E** **, F**). Previous studies reported that incubation of T cells with exogenous hrANX A1 exerted an effect on the activation of Akt/PKB pathways (21). We had confirmed that HA/CD44 axis was able to activate the Akt/mTOR pathway to promote Th1 cell differentiation. As a result, the expression levels of p-mTOR and p-Akt in the naive CD4^+^T cells transfected with NC-lentivirus were significantly increased after the stimulation of HA as compared with the naive CD4^+^T cells transfected with sh*Anxa1*-lentivirus (**Figure 6G**). Taken together, the results from **Figure 6** suggest that HA potentially, through increasing the integration between CD44 and ANX A1, activate the Akt/mTOR pathway, leading to increasing Th1 cells differentiation. Targeting this newly identified HA/CD44/ANX A1/Akt/mTOR signaling may provide novel therapeutic targets for CP/CPPS patients. Furthermore, we infer the mechanisms of the current study in **Figure 6H**.

## 4 Discussion

CP/CPPS is a ubiquitous genitourinary problem. However, current treatment strategies have not yet achieved satisfactory clinical efficacy due to the unclear pathogenesis. In this study, we identified the upregulation of CD44 and its ligand, HA, playing pivotal role in the initiation and progression of CP/CPPS in both mice model and patient levels. Since Th1 cells strongly contribute to the pathogenesis of CP/CPPS (20), here, we found that targeting HA or CD44 can relieve the inflammation and pain severity of EAP mice model by decreasing Th1 cells differentiation. An in-depth mechanism study found that HA can promote the protein interaction of CD44 and ANX A1, followed by the activation of Akt/mTOR pathway, consequently bringing out the enhanced differentiation of Th1 cells. Our research results provide a new target for the clinical treatment of CP/CPPS.

As a cell-surface glycoprotein, CD44 is expressed in many various leukocytes. Consistent with known biological function, many studies had revealed that CD44 is involved in autoimmune disorders and chronic inflammation. Here, we found that inhibiting CD44 expression significantly reduces the severity of prostatic inflammation. Studies had proved that CD44 exerts a critical inflammatory role on T cells. Th1/Th17 cells differentiation was decreased, while Th2 cells differentiation was increased in CD44-deficient mice (15, 16). Recent studies have shown that the Th1-related immune response could induce the occurrence and progression of prostatic inflammation and chronic pelvic pain, and the absence of Th1-related cytokines could significantly reduce EAP susceptibility, while IL-17 was not required for pathological induction and development of chronic pelvic pain (20). In our study, we also found that Th1 cells differentiation in EAP mice model was reduced, which was caused by the downregulated expression of CD44. Moreover, HA, as an important ligand for CD44, exerts proinflammatory effects, such as driving dendritic cell maturation and promoting phagocytosis, antigen presentation, and T-cell activation. Blocking the binding of HA to CD44 significantly reduced the severity of inflammatory diseases, such as rheumatoid arthritis (22), autoimmune diabetes (23), and allergic encephalomyelitis (24), but there is still lack of evidence that supports their role in CP/CPPS. We inhibited the HA expression and found that the severity of prostatic inflammation was significantly relieved. These results were consistent with previous findings that inhibiting the HA synthesis reduces the T-cell migration and modifies the polarization of T cells (17, 18). This evidence preliminarily proved the functional role of HA/CD44 in promoting Th1 cells differentiation driving the pathogenesis of CP/CPPS.

In addition, we found that HA treatment can increase Th1 cells differentiation, accompanied by the activation of the mTOR pathway. Since there is largely unknown relationship between HA stimulation and Akt/mTOR activation, we performed mass spectrum sequencing by pull-down CD44 after HA stimulation. Among several immune-related candidates, we focused on ANX A1. As an endogenous anti-inflammatory factor in the innate immune system, ANX A1 has been suggested to play an important effect in the regulation of adaptive immune response, such as pro- and anti-inflammatory effects. ANX A1^−/−^ mice, as the model for inflammatory diseases, exhibited deregulated inflammatory severity and leukocyte adhesion of arthritis (25). In our study, we found that silencing ANX A1 reversed the HA/CD44-induced Th1 differentiation and Akt/mTOR pathway activation. In addition, studies already proved that ANX A1 could influence the activation of ERK and PKB/Akt signaling (26).

In conclusion, the current study systematically investigates the functional role of HA/CD44 in CP/CPPS and identifies novel mechanisms that HA promotes Th1 cells differentiation. Targeting the HA/CD44/ANX A1/Akt/mTOR signaling represents novel potential therapeutic strategies for CP/CPPS patients.

## Data Availability Statement

The original contributions presented in the study are included in the article/**Supplementary Material**. The data presented in the study are deposited in the CNGBdb repository, accession number CNP0002741, https://db.cngb.org/search/project/CNP0002741/. Further inquiries can be directed to the corresponding authors.

## Ethics Statement

The studies involving human participants were reviewed and approved by the Ethics Committee of the First Affiliated Hospital of Anhui Medical University. The patients/participants provided their written informed consent to participate in this study. The animal study was reviewed and approved by the Animal Center of Anhui Medical University.

## Author Contributions

MZ, XC, and CL: conception and design this study. JC, JM, XLL, XL, and YL: collection and assembly of data. MZ, JC, JC, JM, YL, ZH, and LZ: data analysis and interpretation. JC, JM, MZ, and ZH: manuscript writing. All authors contributed to the article and approved the submitted version.

## Funding

This study was supported by the National Natural Science Foundation of China (81630019, 81870519, and 81802827), Scientific Research Foundation of the Institute for Translational Medicine of Anhui Province (2017ZHYX02), and the Medical and Health Law Research Center Project of Sichuan Province (YF18-19).

## Conflict of Interest

The authors declare that the research was conducted in the absence of any commercial or financial relationships that could be construed as a potential conflict of interest.

## Publisher’s Note

All claims expressed in this article are solely those of the authors and do not necessarily represent those of their affiliated organizations, or those of the publisher, the editors and the reviewers. Any product that may be evaluated in this article, or claim that may be made by its manufacturer, is not guaranteed or endorsed by the publisher.

## Supplementary Material

Supplementary Figure 1 The establishment of EAP mice model and the identification of CD44 in prostate tissues. **(A)** Analysis of histological difference by HE staining. **(B)** Tactile allodynia development in NOD mice from Control and EAP groups. **(C)** Corresponding histopathological score and data analysis according to data in **(A)**. **(D)** The secretion levels of IFN-γ (a), IL-1β (b), and IL-17 (c) in serum of NOD mice from those four groups. **(E)** The expression levels of CD44 in prostate tissues of NOD mice among Control and EAP groups by RNA-sequencing.

Supplementary Figure 2 The detection the expression of OPN, HA, CD44, ANX A1 by WB. **(A)** The expression of important ligands of CD44, OPN and HA, in prostate tissues of mice between Control and EAP groups by WB. **(B)** The expression of p-mTOR, mTOR, p-Akt and Akt of Th1 cells from mice between Control and EAP groups by WB. **(C)** The protein levels of CD44 in CD4^+^T cells after the transfection of sh*Cd44*. **(D)** The protein levels of ANX A1 in CD4^+^T cells after the transfection of sh*Anxa1*.
