# Supplementary figures and images for: HA/CD44 Regulates the T Helper 1 Cells Differentiation by Activating Annexin A1/Akt/mTOR Signaling to Drive the Pathogenesis of EAP
Source: Front Immunol. 2022 May 26;13:875412. doi: 10.3389/fimmu.2022.875412 (PMC9178196; doi:10.3389/fimmu.2022.875412)

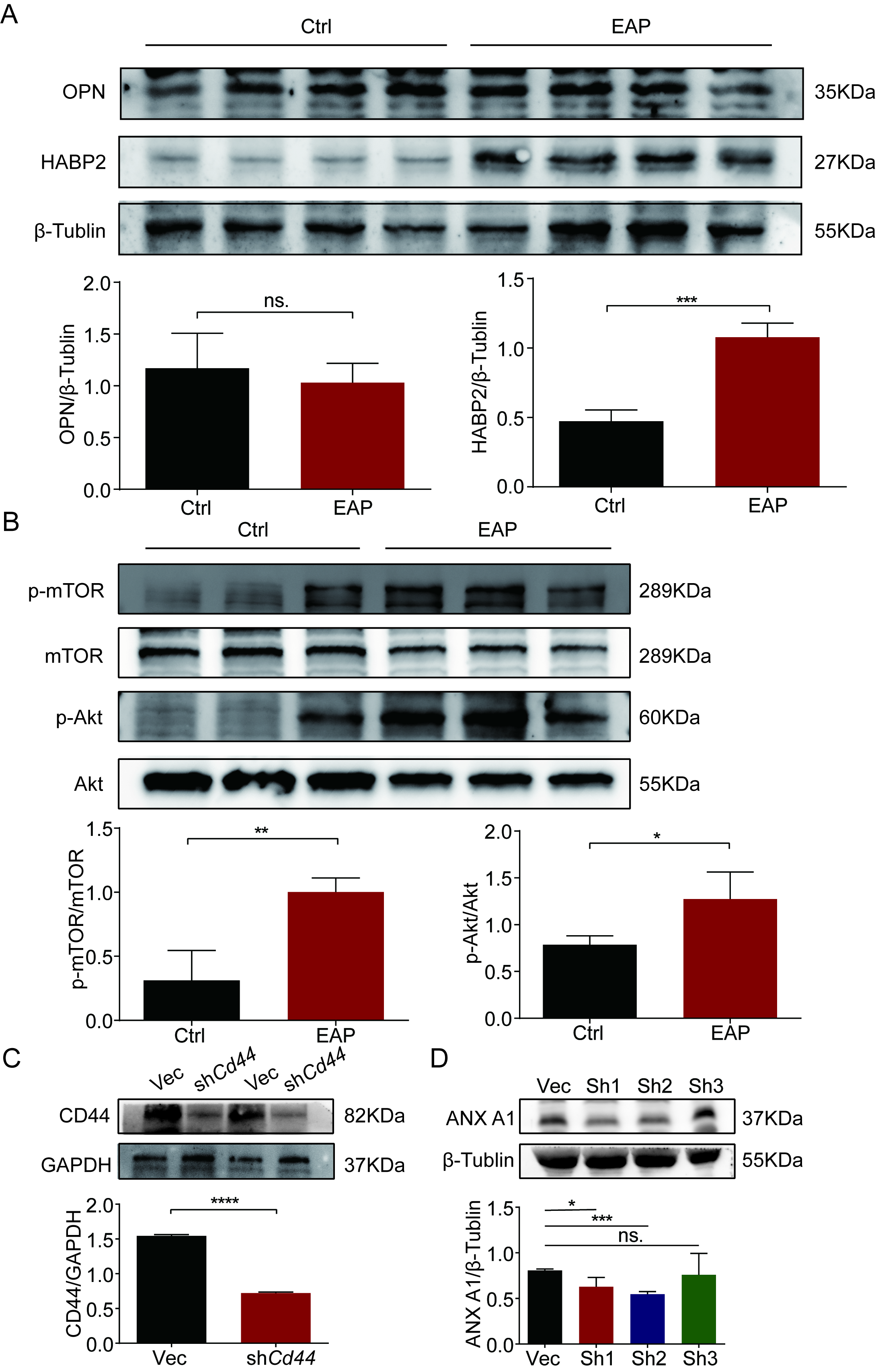

Supplement: Supplementary Figure 2 — The detection the expression of OPN, HA, CD44, ANX A1 by WB. (A) The expression of important ligands of CD44, OPN and HA, in prostate tissues of mice between Control and EAP groups by WB. (B) The expression of p-mTOR, mTOR, p-Akt and Akt of Th1 cells from mice between Control and EAP groups by WB. (C) The protein levels of CD44 in CD4+T cells after the transfection of shCd44. (D) The protein levels of ANX A1 in CD4+T cells after the transfection of shAnxa1. [file Image_2.tif]
