# Supplementary material for: HA/CD44 Regulates the T Helper 1 Cells Differentiation by Activating Annexin A1/Akt/mTOR Signaling to Drive the Pathogenesis of EAP
Source: Front Immunol. 2022 May 26;13:875412. doi: 10.3389/fimmu.2022.875412 (PMC9178196; doi:10.3389/fimmu.2022.875412)
Supplement: Supplementary file 3 [file Table_1.docx]

**Table S1. The expression levels of Annexin A1 among the CP/CPPS-like patients and healthy controls**

|  | **N** | **ANX A1^#^, ng/ml** | ***P* value** |
| --- | --- | --- | --- |
| Healthy control^$^ | 16 | 0.7475 (0.656-1.053) | 0.0223* |
| CP/CPPS-like patients^$^ | 64 | 0.8610 (0.662-2.946) |  |
| NIH-CPSI^$^ |  |  |  |
| < 30 | 47 | 0.8455 (0.662-2.784) | 0.0327* |
| ≥30 | 17 | 1.0450 (0.682-2.946) |  |
| Pain^$^ |  |  |  |
| < 10 | 26 | 0.8205 (0.662-2.500) | 0.0099** |
| ≥ 10 | 38 | 0.9370 (0.780-2.946) |  |

CP/CPPS, chronic prostatitis/chronic pelvic pain syndromes; ANX A1, annexin A1; NIH-CPSI, The National Institutes of Health Chronic Prostatitis Symptom Index; $, median (range)
